# A pediatric case of human herpesvirus 6–associated pityriasis lichenoides et varioliformis acuta

**DOI:** 10.1016/j.jdcr.2025.05.020

**Published:** 2025-06-18

**Authors:** Daniel R. Antohi, Sarah Mazurovsky, Tian Zhu, Hatice Zengin, Bijal Amin, Benedict Wu

**Affiliations:** a Division of Dermatology, Montefiore Medical Center, Albert Einstein College of Medicine, Bronx, New York; b CUNY School of Medicine, New York, New York; c Department of Pathology Montefiore Medical Center, Albert Einstein College of Medicine, Bronx, New York

**Keywords:** HHV-6, human herpesvirus, pityriasis lichenoides et varioliformis acuta, PLEVA

## Introduction

Pityriasis lichenoides et varioliformis acuta (PLEVA) is a rare cutaneous inflammatory disease characterized by diffuse papules at various stages of healing, most often affecting children and adults in their second or third decades of life.^1^ Although the cause of this condition remains largely unknown, PLEVA is associated with certain viruses such as Epstein-Barr virus (EBV), cytomegalovirus, and varicella-zoster virus (VZV). Human herpesvirus (HHV)–7 has also been reported as a potential trigger for PLEVA, suggesting that multiple viral pathogens may serve as possible triggers of this disease.^2^^,^^3^ Although bacterial superantigens (eg, from *Staphylococcus aureus* or *Streptococcus*) and rare drug-induced hypersensitivity reactions have been proposed as additional triggers, many cases remain idiopathic.^3^ Here, we present the first known case of infantile HHV-6–associated PLEVA to highlight that this condition can emerge in young patients with predominant facial and acral lesions and introduce an additional possible viral etiology.

## Case report

**Fig 1:**
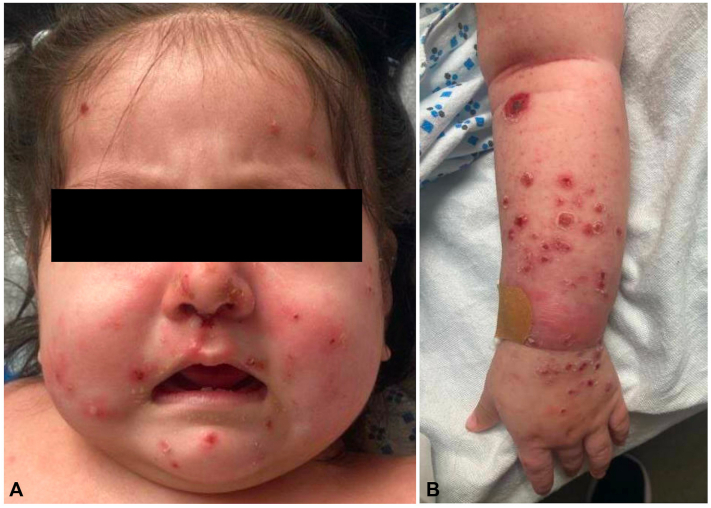
Papulonecrotic lesions in various stages of evolution affecting the (**A**) face and (**B**) extremities.

A 9-month-old female was admitted to the pediatric intensive care unit for a 3-day history of fever, upper respiratory infection symptoms, decreased oral intake, and rash. The dermatology team noted a morbilliform eruption with papulonecrotic lesions in various stages of evolution affecting the face (Fig 1, *A*) and extremities (Fig 1, *B*). The individual lesions consisted of erythematous red to pink papules with collarettes of scale; many of the lesions were herpes-like, leading to a broad differential diagnosis that included reactive inflammatory conditions such as PLEVA, hydroa vacciniforme, and actinic prurigo. Primary infectious conditions, such as a VZV exanthem or rickettsialpox, and neoplastic disorders, including Langerhans cell histiocytosis and EBV-mediated lymphoproliferative disorders, were considered.

**Fig 2:**
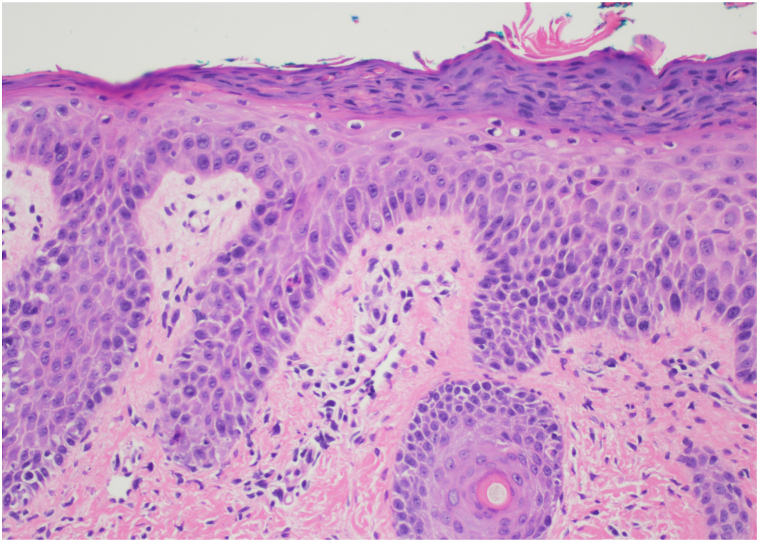
H&E, 20×. Psoriasiform epidermal hyperplasia with parakeratosis subtle vacuolar alteration with scattered necrotic keratinocytes and extravasated red blood cells in the dermis. *H&E*, Hematoxylin and eosin.

**Fig 3:**
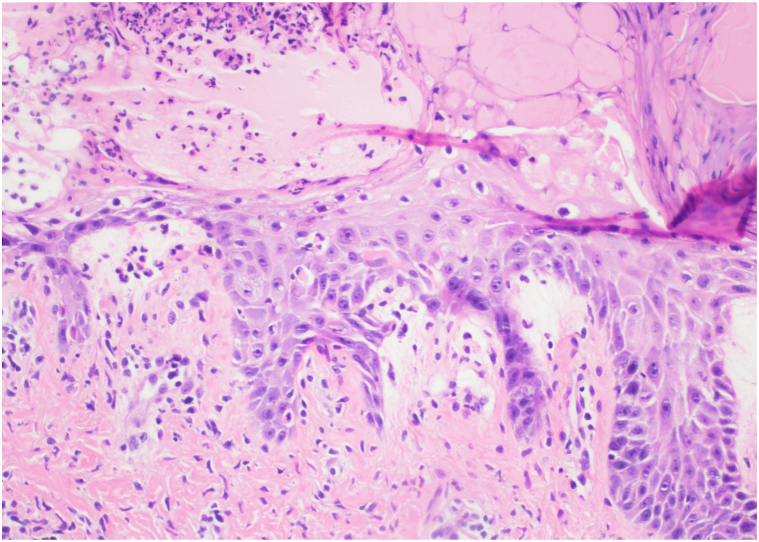
H&E, 20×. Scale and crust containing neutrophils overlying an epidermis with vacuolar alteration and scattered necrotic keratinocytes. There are also extravasated red blood cells in the dermis. *H&E*, Hematoxylin and eosin.

Lesional polymerase chain reaction (PCR) was negative for HHV types 1 to 5 and enterovirus. Plasma VZV PCR and EBV Immunoglobulin M titers were also negative. A lesional punch biopsy showed psoriasiform interface dermatitis with purpura (Fig 2) and an impetiginized ulcer (Fig 3). Following additional workup, the patient was found to have meningitis from HHV-6, confirmed via PCR assays, from the cerebrospinal fluid and plasma. The final diagnosis was PLEVA associated with HHV-6 after clinicopathologic correlation. The patient responded favorably to triamcinolone 0.025% and mupirocin 2% ointments.

## Discussion

PLEVA is considered a benign clonal T-cell disorder in which the clone arises from a subset of T-cells in the skin. The host’s immune response to this clone determines the clinical and histologic findings observed.^4^ Histopathologic findings frequently show dense perivascular and interface lymphocytic infiltrates, with cytotoxic T-cells targeting keratinocytes and causing characteristic necrosis and varioliform scarring. This supports a T-cell–mediated hypersensitivity mechanism, further underscored by reports of T-cell clonality in some patients.^3^

Episodes of PLEVA are classically acute and can recur. PLEVA is linked to multiple etiologies, including drugs, vaccines, and pathogens such as viruses, bacteria, and protozoa.^5^ While viral triggers such as HHVs, EBV, cytomegalovirus, and human immunodeficiency virus are most commonly implicated in PLEVA, bacteria such as *Staphylococcus aureus* and *Streptococcus* species have also been associated.^3^ Reports from several case reports suggest that certain medications may also precipitate this condition.^3^^,^^6^ Agents such as infliximab and iodine contrast have been reported in association with new-onset or exacerbations of PLEVA.^6^^,^^7^ The pathogenesis of drug-induced PLEVA is hypothesized to involve an immune-mediated hypersensitivity reaction in which T-lymphocytes attack keratinocytes, mirroring the pathophysiologic process seen in other etiologies of PLEVA.^3^^,^^6^ Although rarely reported, certain vaccinations have been suggested as potential triggers for the onset of Mucha-Habermann disease, a more fulminant PLEVA variant. For instance, published cases of PLEVA following human papillomavirus vaccination,^8^ anti-tetanus, and diphtheria vaccination^9^ and measles, mumps, and rubella vaccination^10^ highlight the need for clinicians to consider recent immunizations when evaluating new papular eruptions. Our case contributes to the existing literature by describing an unusual case of PLEVA in a young patient presenting with numerous facial and acral lesions incited by HHV-6.

PLEVA treatment aims to address inflammation and mitigate the immune-mediated process. For mild disease, topical corticosteroids may be sufficient to reduce inflammation and pruritus. In our case, a mild topical steroid was sufficient to promote healing of the lesions. Phototherapy may be employed for more extensive involvement, and in recalcitrant or severe cases, systemic options such as methotrexate, cyclosporine, or oral antibiotics have been reported to help induce remission.^3^ Although there is no universally established regimen, most treatments focus on controlling the T-cell–mediated response thought to underlie the condition.

## Conflicts of interest

None disclosed.
